# Emerging Impact of Non-coding RNAs in the Pathology of Stroke

**DOI:** 10.3389/fnagi.2021.780489

**Published:** 2021-11-19

**Authors:** Soudeh Ghafouri-Fard, Zeinab Shirvani-Farsani, Bashdar Mahmud Hussen, Mohammad Taheri, Noormohammad Arefian

**Affiliations:** ^1^Department of Medical Genetics, School of Medicine, Shahid Beheshti University of Medical Sciences, Tehran, Iran; ^2^Department of Cell and Molecular Biology, Faculty of Life Sciences and Technology, Shahid Beheshti University, Tehran, Iran; ^3^Department of Pharmacognosy, College of Pharmacy, Hawler Medical University, Erbil, Iraq; ^4^Institute of Human Genetics, Jena University Hospital, Jena, Germany; ^5^Skull Base Research Center, Loghman Hakim Hospital, Shahid Beheshti University Hospital, Tehra, Iran

**Keywords:** lncRNA, miRNA, stroke, expression, biomarker

## Abstract

Ischemic stroke (IS) is an acute cerebral vascular event with high mortality and morbidity. Though the precise pathophysiologic routes leading to this condition are not entirely clarified, growing evidence from animal and human experiments has exhibited the impact of non-coding RNAs in the pathogenesis of IS. Various lncRNAs namely MALAT1, linc-SLC22A2, linc-OBP2B-1, linc_luo_1172, linc-DHFRL1-4, SNHG15, linc-FAM98A-3, H19, MEG3, ANRIL, MIAT, and GAS5 are possibly involved in the pathogenesis of IS. Meanwhile, lots of miRNAs contribute in this process. Differential expression of lncRNAs and miRNAs in the sera of IS patients versus unaffected individuals has endowed these transcripts the aptitude to distinguish at risk patients. Despite conduction of comprehensive assays for evaluation of the influence of lncRNAs/miRNAs in the pathogenesis of IS, therapeutic impacts of these transcripts in IS have not been clarified. In the present paper, we review the impact of lncRNAs/miRNAs in the pathobiology of IS through assessment of evidence provided by human and animal studies.

## Introduction

**FIGURE 1:**
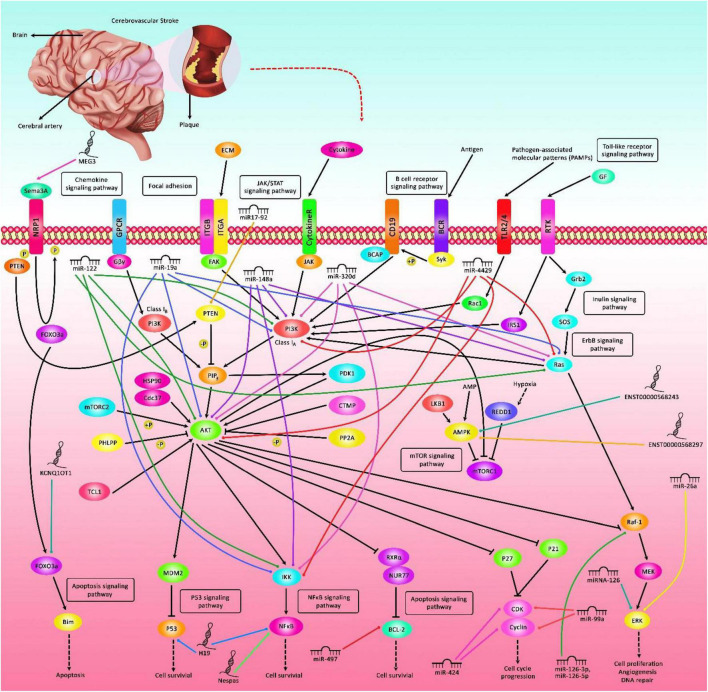
A schematic representation of the interaction between non-coding RNAs and various signaling cascades in ischemic stroke (IS). Differential expression of lncRNAs as well as miRNAs could have an important role in the pathogenesis of IS. Various miRNAs such as miR-19a, miR-122, miR-148a, miR-320d, and miR-4429 via targeting Akt, PI3K, Ras, and IKK could modulate expression of genes leukocytes, thus affecting the course of IS. In addition, miR-497 by regulating the expression levels of Bcl-2 and Bcl-w could induce ischemic neuronal death. Additionally, lncRNA H19 via directly targeting P53 could suppress neurogenesis following IS through p53/Notch1 axis. Furthermore, MEG3 could promote cell survival and reduce cell apoptosis via modulating the expression of Sema3A. Besides, KCNQ1OT1 through modulating the expression of FOXO3 could enhance brain injury and induce autophagy in IS.

Ischemic stroke (IS) is an acute cerebrovascular event with high mortality and morbidity. This disorder is the third most frequent cause of mortality in Western regions of the world (Feigin et al., 2015). Current treatments for IS include thrombolysis, mechanical thromboectomy and neuroprotective therapies (Liaw and Liebeskind, 2020). Although the exact pathophysiologic routes leading to this condition are not entirely clarified, growing evidence from animal and human experiments has exhibited the impact of non-coding transcripts in the pathogenesis of IS (Zhu et al., 2019). These transcripts are highly variable in the terms of size, function, genomic location and conservation, yet in a broad classification they can be categorized based on their size to small versus long non-coding RNAs (lncRNAs). Small non-coding RNAs have some subclasses among them are microRNAs (miRNAs). Both lncRNAs and miRNAs have regulatory impacts on gene expression but via different routes. Being firstly discovered in 1993 in *C. elegans* (Lee et al., 1993), miRNAs comprise an ever-growing type of non-coding RNAs that target specific sequences in the 3′ untranslated regions of genes, then decreasing their expression via mRNA degradation or translation blocking (O’Brien et al., 2018). These transcripts are about 22 nucleotides in length. They can hypothetically target almost any gene in the human genome. However, the extent of miRNA response elements complementarity defines their route of action, i.e., AGO2-dependent cleavage of target transcript or RISC-associated translational suppression (Jo et al., 2015). Besides, a number of miRNAs might influence gene expression at transcriptional and post-transcriptional stages within the nucleus (O’Brien et al., 2018). However, this mode of action has not been fully discovered. The dynamic nature of miRNA-associated gene regulation potentiates them as tools for regulation of gene expression in a cell type/situation-specific mode since several events such as alternative splicing events, polyadenylation state and the presence of cell type-specific RNA binding proteins affect miRNA response elements (O’Brien et al., 2018). LncRNAs are another group of transcripts with fundamental roles in the regulation of gene transcription via several modes including acting as signal, decoy molecules, scaffolds, guide and enhancer transcripts. The chief mode action of lncRNAs is their role in the regulation of transcription in reaction to numerous stimuli through acting as molecular signals (Fang and Fullwood, 2016). Although they generally do not have open reading frame, many of them have similar characteristics with protein-coding genes among them are the presence of 5′ cap, poly A tail and alternative splicing events (Cheng et al., 2005; Derrien et al., 2012). Through participating in chromatin configuration alteration, interaction with chromatin structures, acting as competing endogenous RNAs (ceRNAs) or natural antisense lncRNAs, lncRNAs contribute in the pathogenesis of human disorders (Fang and Fullwood, 2016). Figure 1 depicts the role of a number of non-coding RNAs in the pathobiology of IS through different signaling pathways particularly PI3K/AKT and NF-κβ.

## Human Studies

### Long Non-coding RNAs and Ischemic Stroke

Assessment of expression of lncRNAs has been the focus of numerous studies conducted in human subjects. For instance, a high throughput study has been performed on blood specimens of patients with IS and controls who have been matched with cases in terms of vascular risk factors. The study has revealed differential expression of approximately 300 lncRNAs between IS group and male controls, while 97 lncRNAs have been differentially expressed between IS group and female controls. Notably, some of differentially expressed lncRNAs have been shown to reside in genomic regions formerly recognized as IS risk loci namely lipoprotein, lipoprotein(a)-like 2, ABO blood group, prostaglandin 12 synthase, and α-adducins (Dykstra-Aiello et al., 2016). Another study has reported distinct lncRNAs signatures in peripheral blood mononuclear cells (PBMCs) among patients with IS, transient ischemic attack (TIA) and healthy subjects. Notably, expressions of linc-DHFRL1-4, SNHG15, and linc-FAM98A-3 have been substantially increased in IS patients versus healthy controls and TIA patients. Expression of linc-FAM98A-3 has been returned to normal level by day 7, whereas SNHG15 levels have been continued to be high during the follow-up period, demonstrating the capability of lncRNAs to observe IS dynamics (Deng et al., 2018). Another microarray-based assay has reported up-regulation of 560 and down-regulation of 690 lncRNAs in IS patients versus controls among them have been lncRNAs ENST00000568297, ENST00000568243, and NR_046084. Dysregulated lncRNAs have been predicted to partake in IS pathology by modulating central miRNAs, mRNAs, or IS-associated pathways (Guo et al., 2018). Assessment of lncRNA signature at two time points after IS has revealed differential expression of 3,009 and 2,034 lncRNAs 24 h and 7 days after IS, respectively. These results have shown the impact of IS on lncRNA signature at both the acute and subacute phases. Notably, expression of lncRNAs in the processing and presentation processes of antigens have been increased at 24 h and returned to basal amounts on day 7 following IS. Besides, expressions of inflammatory mediator regulation of TRP channels and GABAergic synapses have been decreased on day 7 following IS (Zhu et al., 2018). Levels of H19 in the circulation of patients with IS have been positively correlated with the National Institute of Health Stroke Scale Scores of the patients in in three time points following stroke attack. Mechanistically, H19 silencing could reduce expression of neurogenesis related proteins. In addition, H19 precludes the development of neurogenesis after IS via p53/Notch1 pathway (Wang et al., 2019a). Another experiment has reported association between H19 and Acute Stroke Treatment (TOAST) subclasses of atherosclerotic patients. Forced over-expression of H19 has enhanced ACP5 expression, increased cell proliferation and blocked cell apoptosis. Up-regulation of H19 has increased the plaque size in the animal model, thus H19 participates in the atherosclerotic processes and surges the risk of IS through increasing ACP5 levels (Huang et al., 2019). RMST is another up-regulated lncRNA in the plasma specimens of IS patients (Hou and Cheng, 2018). A previous study in Chinese Han population has shown over-expression of ANRIL in IS patients parallel with down-regulation of CDKN2A. The rs2383207 and rs1333049 SNPs have been associated with risk of IS in male subjects (Yang et al., 2018). Another study has reported higher levels of ANRIL in patients with the atrial fibrillation (AF) and ischemic stroke compared with AF patients without IS. Serum levels of ANRIL have been correlated with the NIHSS and the mRS scores (Zeng and Jin, 2020). Table 1 gives a summary of human studies reporting elevation of lncRNAs in IS.

**TABLE 1:** Human studies showing elevation of lncRNAs in IS.

lncRNAs	The specimen types	Numbers of clinical specimens	Cell models	Targets/Regulators	Signaling pathways	Function	References
ANRIL	Blood	71 IS patients and 71 normal controls.	−	CDKN2A	−	ANRIL has a role in pathology of IS.	Yang et al., 2018
ANRIL	Serum	132 AF patients with IS and 254 AF without IS	−	−	−	Serum ANRIL is a marker in AF with IS.	Zeng and Jin, 2020
GAS5	Blood	509 IS patients and 668 healthy controls	−	−	−	GAS5 overexpression is associated with increased IS risk.	Zheng et al., 2018
H19		85 IS patients and 85 healthy controls	VSMC and HUVECs	ACP5	−	H19 has enhanced ACP5 expression, increased cell proliferation and blocked cell apoptosis.	Huang et al., 2019
H19	plasma	40 patients with acute ischemic stroke and 25 controls	−	p53	p53/Notch1 pathway	H19 represses neurogenesis following IS via p53/Notch1 axis.	Wang et al., 2019a
H19	Plasma, neutrophils, and lymphocytes	36 patients with anterior circulation ischemia, and 25 normal subjects	BV2 cells	HDAC1	−	H19 induces neuroinflammatory responses.	Wang et al., 2017
KCNQ1OT1	Blood	42 IS patients and 40 healthy controls	N2a	FOXO3	miR−200a/FOXO3/ATG7 pathway	KCNQ1OT1 expression enhanced brain injury and induced autophagy in IS.	Wang et al., 2019a
linc-SLC22A2, linc-OBP2B-1, linc_luo_1172	Blood	133 IS patients and 133 controls	−	−	−	−	Dykstra-Aiello et al., 2016
linc-DHFRL1-4, SNHG15 and linc-FAM98A-3	Blood	206 IS patients, 55 TIA patients and 179 controls	−	−	−	−	Deng et al., 2018
lncRNA-ENST00000568297, lncRNA-ENST00000568243, NR_046084	Blood	50 IS patients and 50 controls	−	BCG5, FOXJ3, MAP3K5	PI3K-Akt, p53 pathway, AMPK pathway	LncRNA-ENST00000568297 and lncRNA-ENST00000568243 Are possible diagnostic biomarkers for IS.	Guo et al., 2018
lnc-CRKL-2, lnc-NTRK3-4	serum	100 AMS patients and 100 healthy controls	−	−	−	These new lncRNAs are markers for the detection of AMS.	Xu et al., 2020
MALAT1	Serum	40 CIS patients and 40 healthy controls	HBMECs	VEGFA	miR-205-5p/VEGFA Pathway	MALAT1 preserves angiogenic properties of HBMECs under OGD/R circumstances.	Gao et al., 2020
MEG3	PBMCs	20 IS patients and 20 controls.	mouse brain neuroma cell line, N2a	miR-424-5p, Sema3A	MAPK	MEG3 enhances cell survival and decreased cell apoptosis.	Xiang et al., 2020
MIAT	Blood	189 IS patients and 189 healthy controls	−	−	−	MIAT is a biomarker for discriminating IS patients from healthy persons.	Zhu et al., 2018
RMST	plasma	10 AIS patients and 10 controls	hippocampal cells	−	−	RMST induces ischemic brain injury and disrupts neurological function.	Hou and Cheng, 2018
SCARNA10, TERC, LINC01481	Blood	10 IS and 5 controls	−	−	−	These LncRNAs play an important role in peripheral immune system changes after IS.	Zhu et al., 2019

Contrary to two mentioned studies in the previous section, Feng et al. have demonstrated decreased levels of ANRIL in plasma specimens of patients with acute IS patients versus controls (Feng et al., 2019). ZFAS1 is another down-regulated lncRNA in IS patients. Moreover, expression of ZFAS1 in patients with large-artery atherosclerosis (LAA) stroke has been lower compared with those with non-LAA stroke and controls. In addition, ZFAS1 expression has been lower in the small vessel occlusion group compared with cardioembolism (Wang et al., 2019b). FLJ23867, H3F3AP6, TNPO1P1 are also among lncRNAs which are down-regulated in IS PBMCs compared with control PBMCs (Zhu et al., 2019). An lncRNA profiling using RNA-seq method and subsequent KEGG pathway and gene ontology (GO) enrichment assays have shown down-regulation of RPS6KA2-AS1 and lnc-CALM1-7 in exosomes retrieved from sera of patients with acute minor IS (Xu et al., 2020). Table 2 gives a summary of human studies that displayed under-expression of lncRNAs in IS.

**TABLE 2:** Summary of clinical investigations reporting under-expression of lncRNAs in IS.

lncRNAs	The specimen types	Numbers of clinical specimens	Function	References
ANRIL	Blood	126 AIS patients and 125 controls	The reduced expression of ANRIL was related with higher risk of IS, higher disease severity and high inflammatory responses in acute IS patients.	Feng et al., 2019
NR_036641, ENST0000079667, ENST00000507442	Blood	133 IS patients and 133 controls	−	Dykstra-Aiello et al., 2016
ZFAS1		176 IS patients and 111 controls	ZFAS1 had an appropriate diagnostic value for large-artery atherosclerosis stroke	Wang et al., 2019b
FLJ23867, H3F3AP6, TNPO1P1	Blood	10 ischemic stroke and 5 controls	These LncRNAs play an important role in peripheral immune system alterations after IS.	Zhu et al., 2019
RPS6KA2-AS1, lnc-CALM1-7	serum	100 AMS patients and 100 healthy controls	These new lncRNAs are biomarkers AMS.	Xu et al., 2020

ANRIL low-expression has been determined as a marker of better recurrence-free survival in of AF patients with IS. Based on the outcomes of Cox regression model, serum levels of ANRIL, NIH Stroke Scale (NIHSS) score, infarct volume, and smoking have been the risk factors for AF with IS (Zeng and Jin, 2020). Another study has reported that down-regulation of ANRIL in acute IS can differentiate these patients from healthy subjects with area under curve (AUC) of 0.759. Besides, expression levels of this lncRNA has been negatively correlated with NIHSS score and high-sensitivity C-reactive protein (hs-CRP), TNF-α and IL-6 concentrations, while being positively correlated with IL-10 concentrations (Feng et al., 2019). Down-regulation of ZFAS1 could predict risk of LAA strokes. Based on the results of receiver operating characteristic curve, ZFAS1 has 89.39% sensitivity in distinguishing LAA stroke patients from controls (Wang et al., 2019b). Another biomarker discovery study in PBMCs of IS patients has demonstrated AUC values of 0.73, 0.74, and 0.69 for ENST00000568297, ENST00000568243 and NR_046084, respectively (Deng et al., 2018). Moreover, MIAT levels in IS patients have been remarkably increased in correlation with NIHSS scores, mRS, hs-CRP and infarct size. Based on the results of ROC (receiver operating characteristic) curves, MIAT has been suggested as a possible marker for distinguishing IS patients from the healthy subjects with AUC value of 0.842. Moreover, patients with over-expression of MIAT had a comparatively poor prognosis. Multivariate analysis has shown the potential of MIAT as an independent prognostic biomarker of functional outcome and mortality of IS (Zhu et al., 2018). Table 3 gives a brief review of investigations that reported diagnostic/prognostic role of lncRNAs in IS.

**TABLE 3:** Diagnostic/prognostic role of lncRNAs in stroke.

Samples	Area under curve	Sensitivity	Specificity	Kaplan-Meier analysis	Univariate cox regression	Multivariate cox regression	References
Blood specimens from 126 AIS patients and 125 controls	0.759 for ANRIL	72.2% for ANRIL	71.2% for ANRIL	−	−	−	Feng et al., 2019
Blood specimens from 206 Ischemic stroke patients in the acute phase, 55 transient ischemic attack patients and 179 healthy controls	0.711 for linc-DHFRL1-4, 0.756 for SNHG15, 0.659 for linc-FAM98A-3	0.687 for linc-DHFRL1-4, 0.594 for SNHG15, 0.594 for linc-FAM98A-3	0.719 for linc-DHFRL1-4, 0.844 for SNHG15, 0.688 for linc-FAM98A-3	−	−	−	Deng et al., 2018
Blood specimens from 50 patients with IS and 50 controls	0.733 for ENST00000568297, 0.743 for ENST00000568243, 0.690 for NR_046084	64.8% for ENST00000568297, 70.5% for ENST00000568243, 61.5% for NR_046084	63.6% for ENST00000568297, 69.5% for ENST00000568243, 69.2% for NR_046084	−	−	−	Guo et al., 2018
176 IS patients and 111 controls	0.727 for ZFAS1	89.39 for ZFAS1	48.65 for ZFAS1	−	−	ZFAS1 low expression was associated with risk of LAA strokes.	Wang et al., 2019b
Blood specimens from 71 IS patients and 71 normal controls.	0.642 for ANRIL	0.663 for ANRIL	0.538 for ANRIL	−	−	−	Yang et al., 2018
Blood specimens from 189 IS patients and 189 healthy controls	0.842 for MIAT	74.1% for MIAT	80.4% for MIAT	Patients with over-expression of MIAT had a higher mortality compared with the low-MIAT patients. High MIAT was associated with poor prognosis.	Elevated MIAT Has been associated with IS.	MIAT was an independent prognostic indicator of functional consequences and mortality.	Zhu et al., 2018
Serum specimens from 132 AF patients with IS and 254 AF without IS	0.826 for ANRIL	76.6% for ANRIL	81.4% for ANRIL	Patients with lower lncRNA ANRIL expression had higher relapse-free survival compared with the high−expression group.	Serum ANRIL expression, NIHSS score, infarct size, and smoking were the risk factors for AF with IS.	Serum ANRIL expression and smoking were independent risk factors for AF with IS.	Zeng and Jin, 2020

### MicroRNAs and Stroke

Expression of miR-205-5p has been surged in the serum specimens of CIS patients and human brain microvascular endothelial cells under oxygen glucose deprivation/re-oxygenation. Besides, this condition has interfered with the tube formation of human brain microvascular endothelial cells. miR-205-5p knock-down has enhanced proliferation and angiogenic capacity of endothelial cells to resist oxygen glucose deprivation/re-oxygenation injury (Gao et al., 2020). The relationship between upper limb recovery after IS and miRNA signature has been assessed by another group. Authors have discovered lower levels of miR-371-3p, miR-524, miR-520g, miR-1255A, miR-453, and miR-583, while upper levels of miR-941, miR-449b, and miR-581 in good recover group compared with poor recovery group. These miRNAs have been shown to congregate on pathways related with axon guidance, developmental processes and carcinogenesis (Edwardson et al., 2018b). Expression of let-7e-5p has also been shown to be elevated in IS patients compared with control subjects. Over-expression of let-7e-5p has been associated with elevated probability of IS. This miRNA has been suggested to influence expression of four genes enriched in the MAPK pathway including CASP3 and NLK (Huang et al., 2016). miRNA levels might also distinguish IS patients from those with hemorrhagic stroke (HS). Leung et al. have demonstrated higher median plasma levels of miR-124-3p in acute phase of HS patients compared with similar phase of IS, while miR-16 had the opposite trend. Both miRNAs have been suggested as diagnostic markers for discrimination of HS from IS (Leung et al., 2014). A high throughput miRNA profiling in IS has reported differential expression of 115 miRNAs between IS cases and healthy controls. These transcripts have been linked with axon guidance, glioma, MAPK, mTOR and Erb-B signaling pathways. miR-32-3p, miR-106-5p, and miR-532-5p have been the first ranked ones (Li et al., 2015). Table 4 provides the summary of researches which reported elevation of miRNAs in IS.

**TABLE 4:** Summary of human studies reporting elevation of miRNAs in IS.

microRNA	The specimen types	Numbers of clinical specimens	Cells	Targets/Regulators	Signaling pathways	Function	References
let-7e-5p	Blood	302 IS patients and 302 healthy controls	U937 cell line	CASP3 and NLK	MAPK signaling pathway	Let-7e-5p might be a useful noninvasive marker for the diagnosis of IS.	Huang et al., 2016
miR-145	Blood	32 IS patients and 18 healthy controls	−	KLF4/5	−	MiR-145 might serve as a useful biomarker and therapy for IS.	Gan et al., 2012
miR-363, miR-487b	Blood	24 AIS patients and 24 control	−	MAP2K4	toll-like receptor signaling pathway	These miRNA may regulate leukocyte gene expression.	Jickling et al., 2014
miR-125b-2, miR-27a, miR-422a, miR-488 and miR-627	Blood	169 stroke patients, 24 healthy controls, and 94 individuals with metabolic syndrome	−	−	−	These miRNAs may serve as potential diagnostic biomarkers for IS.	Sepramaniam et al., 2014
miR-9-5p, miR-9-3p, miR-107, miR-124-3p, and miR-128-3p	CSF	21 IS patients and 21 controls	−	−	−	These miRNAs show the ischemia-related brain damage.	Sørensen et al., 2017
miR-17-5p, miR-20b-5p, miR-27b-3p, miR-93-5p	Extracellular Vesicle in blood	34 non-stroke and 139 stroke patients	−	−	stress/hypoxia and repair pathways	These miRNAs profile shows the development of cerebral SVD.	van Kralingen et al., 2019
hsa-miR-4656, hsa-miR-432, hsa-miR-503, hsa-miR-376c, hsa-miR-130a-3p and hsa-miR-487b	PBMCs	20 IS patients and 19 healthy controls	−	TGFB3, CELSR2 and ITM2C	−	These miRNAs regulate immune responses.	Bam et al., 2018
let-7b	Plasma	197 IS patients and 50 controls	−	−	−	Let-7b might serve as a useful noninvasive marker for the diagnosis of IS.	Long et al., 2013
miR-124-3p and miR-16	Plasma	74 IS and 19 HS	−	−	−	miR-124-3p and miR-16 Expression levels may be the potential circulating biomarker to distinguish hemorrhagic stroke and IS.	Leung et al., 2014
miR-222, miR-218, and miR-185	Plasma	106 AIS patients and 110 controls	−	−	−	These miRNAs might serve as promising and independent biomarkers for risk of AIS.	Jin and Xing, 2017
hsa-miR-106b-5P, hsa-miR-4306	Plasma	136 AIS patients and 116 healthy controls	−	−	−	Enhanced expression of hsa-miR-106b-5P and hsa-miR-4306 in plasma may be novel biomarkers for the early detection of AIS.	Wang et al., 2014
miR-16	Plasma	40 HACI patients and 30 healthy controls.	−	−	−	The high expression of miR-16 in plasma were related to TOAST and OCSP criteria.	Tian et al., 2016
miR-143-3p, miR-125b-5p, miR-125a-5p	Plasma	200 IS patients and 100 healthy controls	−	−	−	A combination of miR-125a-5p, miR-125b-5p, and miR-143-3p might have clinical utility as an early diagnostic biomarker.	Tiedt et al., 2017
miR-125b-5p and miR-206	Plasma	94 AIS patients with or without endovascular treatment	−	−	−	miR-125b-5p and miR-206 levels are related with stroke severity.	van Kralingen et al., 2019
miR-371-3p and miR-520g	Plasma	27 IS patients	−	−	−	These miRNAs are markers of neural repair.	Edwardson et al., 2018a
miR-205-5p	Serum	40 CIS patients and 40 healthy controls	HBMECs	MALAT1	-	miR-205-5p inhibits proliferation of endothelial cells.	Gao et al., 2020
miR-15a, miR-16, and miR-17-5p	Serum	106 AIS patients and 120 healthy controls	−	−	−	Combination of miR-15a, miR-16, and miR-17-5p may be a potential AIS biomarker.	Wu et al., 2015
miR-15a and miR-16	Serum	20 CLI patients, 122 T2D+ CLI patients, and 43 healthy controls	Circulating proangiogenic cells (PACs), VSMCs, and pericytes	VEGF-A and AKT3	AKT signaling pathway	miR-15a/16 induces PAC survival and migration, and increases migratory capacity of PACs	Spinetti et al., 2013
miR-145	Serum	146 AIS patients and 96 control	−	−	−	MiR-145 might serve as a useful biomarker and therapy for IS.	Jia et al., 2015
miR-9 and miR-124	Serum	65 AIS patients and 66 control	−	−	−	Serum exosomal miR-9 and miR-124 are markers for AIS.	Ji et al., 2016
miR-223	Serum	50 AIS patients and 33 control	−	−	−	Exosomal miR-223 levels are associated with acute IS.	Chen et al., 2017
miR-146b	Serum	128 AIS patients and 102 control	−	−	−	Elevated serum miR-146b expression might be a potential biomarker for AIS evaluation.	Chen et al., 2018b
miR32-3p, miR-106b-5p, miR-423-5p, miR-451a, miR-1246, miR-1299, miR-3149, and miR-4739	Serum	117 AIS patients and 82 healthy controls	−	−	−	These miRNAs may serve as potential diagnostic biomarkers for IS.	Li et al., 2015
miR-23b-3p, miR-29b-3p, miR-181a-5p and miR-21-5p	Serum	177 IS, 81 TIA patients and 42 controls.	−	−	−	Enhanced expression of miR-23b-3p, miR-29b-3p and miR-21–5p might distinguish between IS and TIA.	Wu et al., 2017
PC-3p-57664, PC-5p-12969, hsa-miR-122-5p and hsa-miR-211-5p	Serum	34 IS patients and 11 healthy controls. postmortem specimens from 10 IS brains and 10 control brains	lymphoblastoid cell line	−	−	These miRNAs are biomarkers for IS.	Vijayan et al., 2018
let-7e	serum and cerebral spinal fluid	72 IS patients and 51 healthy controls	−	−	−	let-7e Expression levels in serum may be the potential circulating biomarker for the acute stage of ischemic stroke.	Peng et al., 2015

Blood amounts of miR-30a and miR-126 have been substantially decreased in all assessed patients with IS until 24 weeks. Circulating let-7b has been decreased in patients with LAA compared with healthy subjects, while circulating let-7b have been higher in patients with other kinds of IS until 24 weeks. Notably, aberrant miRNAs levels have been resolved 48 weeks after IS onset in all patients. Authors have suggested that miR-30a might affect IS through modulation of RhoB and beclin-1. Moreover, miR-126 and let-7 can contribute in this process through modulation of VCAM-1 and inflammatory responses, respectively (Long et al., 2013). Another investigation has demonstrated that miRNA signature reveal not only the chronological development of IS but also the specific reasons for development of IS. Authors have suggested a 32-miRNA panel that can distinguish stroke etiologies during the acute phase. Moreover, miR-125b-2^∗^, miR-27a^∗^, miR-422a, miR-488, and miR-627 have been constantly dysregulated in acute stroke independent of age or severity or underlying metabolic background (Sepramaniam et al., 2014). Table 5 provides outcome of human studies reporting down-regulation of miRNAs in IS.

**TABLE 5:** Summary of human studies reporting down-regulation of miRNAs in IS.

microRNA	The specimen types	Numbers of clinical specimens	Cell line	Targets/Regulators	Signaling pathways	Function	References
miR-145		−	Primary astrocytes from rats	AQP4	−	miR-145 protects astrocytes from damage.	Zheng et al., 2017
miR-122, miR-148a, let-7i, miR-19a, miR-320d, miR-4429	Blood	24 AIS patients and 24 control	−	GFR, RAS, PI3K, AKT, IKK	NF-κβ signaling	These miRNA may regulate leukocyte gene expression in IS.	Jickling et al., 2014
miR-574-3p	Blood	55 chronic stroke patients and 2360 controls	−	DBNDD2 and ELOVL1	neurometabolic and chronic neuronal injury response pathways	miR-574-3p has a role in regulating chronic brain and systemic cellular response to stroke	Salinas et al., 2019
miRNA-660-5p	Extracellular Vesicle in blood	34 non-stroke and 139 stroke patients	−	−	−	This miRNA associated with pathophysiology of IS	van Kralingen et al., 2019
hsa-miR-874-3p	PBMCs	20 IS patients and 19 healthy controls	−	IL12A and IL12B	−	hsa-miR-874-3p involves in the immune system alteration during IS pathophysiology	Bam et al., 2018
miR-30a and miR-126	Plasma	197 IS patients and 50 controls	−	−	−	miR-30a and miR-126 are markers of IS.	Long et al., 2013
miR-126, miR-130a, and miR-378	Plasma	106 AIS patients and 110 controls	−	−	−	These miRNAs might serve as promising and independent biomarkers for risk of AIS.	Jin and Xing, 2017
hsa-miR-320e, hsa-miR-320d	Plasma	136 AIS patients and 116 healthy controls	−	−	−	Reduced expression of hsa-miR-320e and hsa-miR-320d is marker for early detection of AIS.	Wang et al., 2014
let-7i-3p and miR-23a-3p	Plasma	10 AIS patients and 10 healthy controls	−	−	−	These miRNAs associated with the peculiarities of clinical manifestations of IS	Zhanin et al., 2018
miR-195	Plasma	96 AIS patients	C57BL/6 mice, BV2 microglial cells and HEK293T cells	CX3CL1 and CX3CR1	CX3CL1/CX3CR1 signaling pathway	miR-195 has neuroprotective roles.	Guang et al., 2019
miR-449b, miR-519b, miR-581, miR-616, miR-892b, miR-941, miR-1179, miR-1292, and miR-1296	Plasma	27 IS patients	−	−	−	These miRNAs show neural repair.	Edwardson et al., 2018a
miR23a and miR-221	Serum	146 AIS patients and 96 control	−	−	−		Jia et al., 2015
miR-124, miR-9	Serum	31 AIS patients and 11 control	−	MMP-9	−	Serum miR-124, miR-9 inhibit neuroinflammation and brain injury.	Liu et al., 2015
miR-224-3p, miR377-5p, miR-518b, miR-532-5p, and miR-1913	Serum	117 AIS patients and 82 healthy controls	−	−	−	These miRNAs in serum may be markers for IS.	Li et al., 2015
miR-146a	Serum	44 IS patients and 22 controls	−	−	−	miR-146a was decreased in patients with more severe conditions.	Kotb et al., 2019
miR-1228-5p, miR-1268a, miR-1268b, miR-4433b-3p, miR-6090, miR-6752-5p, and miR6803-5p	Serum	86 IS patients and 45 controls	−	−	−	These miRNAs forecast the risk of cerebrovascular disorder before the onset of IS.	Sonoda et al., 2019
hsa-miR-22–3p, PC-3p-32463, hsa-miR-30d-5p and hsa-miR-23a-3p	Serum	34 IS patients and 11 healthy controls. postmortem specimens from 10 IS brains and 10 control brains	lymphoblastoid cell line	−	−	These miRNAs could be used as biomarkers for IS.	Vijayan et al., 2018

Diagnostic and prognostic role of miRNAs have been appraised in IS. Elevated serum amounts of miR-15a, miR-16, and miR-17-5p in acute IS patients could be used as diagnostic markers. Based on the multivariate logistic regression analysis, serum miR-17-5p levels could discriminate the presence of acute IS. miR-15a, miR-16, and miR-17-5p had AUC values of 0.698, 0.82, and 0.784, respectively. Combination of three miRNAs increased the AUC value to 0.845 (Wu et al., 2015). ROC curve analysis has revealed AUC values of 0.91, 0.91, 0.92, and 0.93 for plasma miR-30a levels, at 24 h, 1, 4, and 24 weeks, respectively. These values have been 0.93, 0.92, 0.92, and 0.91 for miR-126 at these time points, respectively. Taken together, miR-30a, miR-126 and let-7b can be suitable biomarkers for IS (Long et al., 2013). Expression levels of miR-145 and miR-210 have been remarkably elevated in IS patients with robust AUC values of 0.90 and 1.0, respectively. Yet, dysregulation of miR-145 and miR-210 has not been exclusive for the acute phase as they have been also up-regulated in recovery phase (Sepramaniam et al., 2014). Table 6 provides summary of studies reporting diagnostic/prognostic role of miRNAs in IS.

**TABLE 6:** Diagnostic/prognostic role of miRNAs in IS.

Sample number	Area under curve	Sensitivity	Specificity	Kaplan-Meier analysis	Univariate cox regression	Multivariate cox regression	References
Plasma specimens from 197 IS patients and 50 controls	0.91 for miR-30 0.92 for miR-126 0.93 for let-7b	80% for miR-30, 84% for miR-126, 84% for let-7b	94% for miR-30, 92% for miR-126, 92% for let-7b	−	−	−	Long et al., 2013
serum and cerebral spinal fluid specimens from 72 IS patients and 51 healthy controls	0.86 for let-7e	82.8%	73.4%	−	−	−	Peng et al., 2015
Blood specimens from 302 IS patients and 302 healthy controls	0.82 for let-7e-5p	−	−	−	−	−	Huang et al., 2016
Serum specimens from 106 AIS patients and 120 healthy controls	0.698 for miR-15a, 0.82 for miR-16, and 0.784 for miR-17-5p	−	−	−	−	Serum miR-17-5p is an independent marker for AIS.	Wu et al., 2015
Plasma specimens from 74 IS and 19 HS	0.70 for miR-124-3p, 0.59 for miR-16	68.4% for miR-124-3p, 94.7% for miR-16	71.2% for miR-124-3p, 35.1% for miR-16	−	−	NIHSS, platelet count and the plasma levels of miR-124-3p were significant predictors of HS.	Leung et al., 2014
Plasma specimens from 106 AIS patients and 110 controls	0.767 for combined miRNAs	87.7% for combined miRNAs	54.5% for combined miRNAs	−	miR-126, miR-130a, miR-378, miR-222, miR-218, and miR-185 were predicting factors for risk of AIS.	miR-126 and miR-130a were protective factors for AIS. miR-222, miR-218, and miR-185 were risk factors for AIS.	Jin and Xing, 2017
Serum specimens from 146 AIS patients and 96 control	0.896 for miR-145, 0.816 for miR-23a, 0.819 for miR-221	−	−	−	−	−	Jia et al., 2015
Serum specimens from 65 AIS patients and 66 control	0.8026 for miR-9, 0.6976 for miR-124	−	−	−	−	−	Ji et al., 2016
Serum specimens from 50 AIS patients and 33 control	0.859 for miR-223	84.0% for miR-223	78.8% for miR-223	−	−	Circulating exosomal miR-223 is risk factor for IS.	Chen et al., 2017
Serum specimens from 128 AIS patients and 102 control	0.863 for combination of hs-CRP and miR-146b	−	−	−	−	−	Chen et al., 2018b
Blood specimens from 169 stroke patients, 24 healthy controls, and 94 individuals with metabolic syndrome	0.95 for miR-125b-2, 0.89 for miR-27a, 0.92 for miR-422a, 0.87 for miR-488, 0.84 for miR-627	−	−	−	−	−	Sepramaniam et al., 2014
Plasma specimens from 136 AIS patients and 116 healthy controls	0.962 for hsa-miR-106b-5P; 0.952 for hsa-miR-4306; 0.981 for hsa-miR-320e; 0.987 for hsa-miR-320d	−	−	−	−	−	Wang et al., 2014
Plasma specimens from 40 HACI patients and 30 healthy controls.	0.775 for miR-16	69.7% for miR-16	87% for miR-16	−	−	Patients with higher expression of MiR-16 were associated with poor prognosis.	Tian et al., 2016
Plasma specimens from 200 IS patients and 100 healthy controls.	0.93 for combination of miR-143-3p, miR-125b-5p, and miR-125a-5p	85.6% for combination of miR-143-3p, miR-125b-5p, and miR-125a-5p	76.3% for combination of miR-143-3p, miR-125b-5p, and miR-125a-5p	−	−	−	Tiedt et al., 2017
Serum specimens from 177 IS, 81 TIA patients and 42 controls.	0.883 for combination of miR-23b-3p, miR-29b-3p, miR-181a-5p and miR-21–5p	−	−	−	miR-23b-3p, miR-29b-3p and miR-21–5p levels were independently associated with IS. miR-23b-3p, miR-29b-3p and miR-181a-5p levels were associated with TIA.	Enhanced miR-23b-3p, miR-29b-3p, miR-181a-5p and miR-21–5p levels were closely associated with IS, and enhanced miR23b-3p, miR-29b-3p and miR-181a-5p levels were associated with TIA.	Wu et al., 2017
Serum specimens from 86 IS patients and 45 controls	0.95 for combination of miR-1268b, miR-4433b-3p, and miR-6803-5p	84% for combination of miR-1268b, miR-4433b-3p, and miR-6803-5p	98% for combination of miR-1268b, miR-4433b-3p, and miR-6803-5p	−	−	−	Sonoda et al., 2019
Plasma specimens from 94 AIS patients with or without endovascular treatment	0.735 for miR125b-5p	86.36% for miR125b-5p	55.36% for miR125b-5p	−	−	Higher expression of miR125b-5p associated with an unfavorable outcome.	van Kralingen et al., 2019

### Animal Studies

Investigations in animal models of IS have provided valuable data about the mechanisms of involvement of lncRNAs/miRNAs in IS and possible application of targeted therapies against these transcripts. For instance, expression of RMST has been elevated in primary hippocampal neurons exposed with oxygen-glucose deprivation and in animal models of IS induced by middle cerebral artery occlusion (MCAO). RMST silencing has amended brain injury in the mentioned animal model and attenuated hippocampal neuron defects (Hou and Cheng, 2018). H19 is another up-regulated lncRNA in animal models of IS whose silencing has diminished the size of brain tissue damage following middle cerebral artery obstruction and reperfusion and ameliorated the neurological defects. Mechanistically, H19 silencing could reduce expression of neurogenesis related proteins. Taken together, H19 precludes the development of neurogenesis after IS via p53/Notch1 pathway (Wang et al., 2019a). A throughput miRNA sequencing in infarcted brain regions after regional cerebral ischemia has shown up-regulation of 20 miRNAs while down-regulation of 17 miRNAs in the infarct area among them have been miR-211-5p, miR-183-5p, miR-182, and miR-96-5p which have been functionally related with some important pathways in the neurons (Duan et al., 2019). Table 7 summarizes the data regarding the roles of up-regulated non-coding RNAs in the pathogenesis of IS as revealed by animal studies.

**TABLE 7:** Summary of animal studies which displayed elevation of lncRNAs and miRNAs in stroke.

lncRNAs/miRNAs	Animal models	Cells	Targets/Regulators	Signaling	Function	References
RMST	MCAO mouse model	hippocampal cells	−	−	RMST induces ischemic brain injury and disrupts neurological function.	Hou and Cheng, 2018
GAS5	brain tissues of C57BL/6 J mice	−	miR-137	Notch1 signaling pathway	GAS5 is a ceRNA for miR-137 to control Notch1.	Chen et al., 2018a
Nespas	brain tissues of C57BL/6 J mice	Mouse BV2 microglial cells	TAK1	NF-κB signaling	Nespas induces Neuroinflammation Through inhibiting NF-κB Activation	Deng et al., 2019
MALAT1	brain cortex of C57BL/6 J mice	cortical neurons of mice	Beclin1, miR-30a	−	MALAT1 induces ischemic injury and autophagy.	Guo et al., 2017
H19	C57BL/6 J mice	−	miR-675, IGF1R, pS6 kinase	IGF1 signaling pathway, mTOR pathway	H19 knockdown mice indicated amelioration on the performance of a skilled, cortical dependent motor task.	Wang et al., 2020b
Maclpil	C57BL/6 mice	−	LCP1	−	Maclpil regulates the migration of macrophage and phagocytosis by LCP1.	Wang et al., 2020b
MEG3	C57BL/6 J mice	N2a cell	miR-21	miR-21/PDCD4 pathway	MEG3 promotes ischemic damage and disrupts overall neurological levels.	Zheng et al., 2017
MALAT1	C57BL/6J mice	Mouse BMECs and N2A cells	Bim and E-selectin	apoptotic pathways	Malat1 expression reduced ischemia-induced endothelial cell death *in vitro*	Zhang et al., 2017b
MEG3	SD rats	−	BDNF, NGF and bFGF	Wnt/β-catenin signaling pathway	MEG3 reduced nerve growth and enhanced neurological damage.	You and You, 2019
MEG3	SD rats	rat brain microvascular endothelial cells	NOX4	p53/NOX4 pathway	MEG3 was an important regulator of apoptosis.	Zhan et al., 2017
ANRIL	Wistar rats	neural cells	VEGF	NF-κB signaling pathway	ANRIL increases VEGF and induces angiogenesis.	Zhang et al., 2017a
H19	Wistar rats	Neural stem cell (NSC)	SUZ12, EZH2, miR-675	oxidative response, NF-κβ signaling	H19 expression induces the proliferation and neuronal differentiation of NSCs.	Wang et al., 2020b
H19	C57BL/6J mice	−	p53	p53/Notch1 pathway	H19 represses neurogenesis after IS.	Wang et al., 2019a
MALAT1	C57BL/6 J mice	Primary astrocytes	AQP4, miR-145	−	MALAT1 induced cerebral ischemia-reperfusion damage.	Wang et al., 2020a
H19, Lnc-EF094477 and LncBC090003	Wistar rats	Neural progenitor cells (NPCs)	−	−	H19 regulated post-stroke neurogenesis.	Liu et al., 2019
GAS5	C57BL/6 mice	293 T	MAP4K4	−	GAS5 induses neuron cell apoptosis and nerve injury in ischemic stroke through inhibiting DNMT3B-dependent MAP4K4 methylation	Deng et al., 2020b
MEG3	MCAO rats	OGD/R-treated neurocytes	miR-485 and AIM2	MEG3/miR-485/AIM2 axis	MEG3 induces cerebral ischemia reperfusion injury through elevating pyroptosis by targeting miR-485/AIM2 axis	Liang et al., 2020
miR-211-5p, miR-183-5p, miR-182 and miR-96-5p	Brain of 10 Rat MCAO model and 10 controls	−	PPFIA1, SLC7A1, NTRK2, KDM48	Ras, cGMP-PKG and phospholipase D signaling pathways	These miRNAs may control cell proliferation and apoptosis via the cGMP-PKG signaling pathway.	Duan et al., 2019
miR-669c-3p	Primary cortical neuron cultures and Primary microglial cultures from C57BL/6 J neonatal mice	N2a cell line	MyD88	toll-like receptor signaling pathway	miR-669c overexpression modulates the inflammatory responses.	Kolosowska et al., 2020
miR-3473b	brain tissues from CD-1 mice	BV2 microglial cells	SOCS3	−	The expression of miR-3473b activates microglial and the inflammation and induces neuroinflammation.	Wang et al., 2018
miR-26a	Brain tissues from 48 SD rats	−	HIF-1a and VEGF	PI3K/AKT and MAPK/ERK pathway	miR-26a controls cell proliferation and angiogenesis.	Liang et al., 2018b
miR17-92	C57BL/6J mice	SVZ neural progenitor cells	PTEN	Shh signaling pathway	miR17-92 induces the proliferation and viability of SVZ neural progenitor cells.	Liu et al., 2013
miR-92a	mice	human endothelial cells	integrin subunit alpha5	−	miR-92a increased angiogenesis and functional recovery of injured tissue.	Bonauer et al., 2009
miR-497	C57/B6 mice	mouse neuroblastoma (N2A) cells	bcl-2 and bcl-w	ischemia-induced cell death signaling pathway	miR-497 induces ischemic neuronal death.	Yin et al., 2010
miR-130a	Brain tissue from SD rats	neurons	XIAP	−	miR−130a inhibits the proliferation, viability, and differentiation of NSCs.	Deng et al., 2020a
miR-125b	plasma and brain tissue specimens from 50 SD rats	PC-12 cell line	CK2α	CK2α/NADPH Oxidase Signaling pathway	miRNA-125b increases cerebral ischemia injury.	Liang et al., 2018b
miR-223-5p	primary cortical neurons from Wistar rat, SD rats	cortical neurons	NCKX2	−	miR-223-5p amended ischemic damage and enhanced neurological function.	Cuomo et al., 2019
miR-155	C57BL/6 mice	endothelial cells	Dhx40, Dync1i1, Zfp652, Agtr1a	proangiogenic signaling pathway	miR-155 reduces blood flow and cerebral microvasculature.	Caballero-Garrido et al., 2015
miR-155	C57BL/6 mice	−	−	−	Mir-155 promotes ischemia/reperfusion induced brain injury and hemorrhagic transformation	Suofu et al., 2020

Meg3 is a down-regulated lncRNA after IS. Up-regulation of Meg3 has inhibited functional recovery and diminished capillary mass after IS. On the other hand, its silencing has amended brain lesions and enhanced angiogenesis after IS. Meg3 exerts these functions through inhibiting notch pathway (Liu et al., 2017). Expression of lncRNA-1810034E14Rik has also been down-regulated in LPS-exposed or oxygen-glucose deprivation-induced microglial cells. Up-regulation of 1810034E14Rik has reduced the infarct volume, ameliorated brain injury in MCAO model and decreased production of inflammatory cytokines both in the animal model and in microglial cells. Besides, 1810034E14Rik up-regulation could block the induction of microglial cells and suppress p65 phosphorylation of p65 (Qu et al., 2019). The above-mentioned examples indicate that down-regulation of lncRNAs in IS might be a compensative mechanism for amelioration of neuron damage or can be directly participate in the pathogenic mechanisms during IS. Table 8 summarizes the data regarding the roles of down-regulated non-coding RNAs in the pathogenesis of IS as revealed by animal studies.

**TABLE 8:** Summary of animal studies which displayed down-regulation of lncRNAs and miRNAs in stroke.

lncRNAs/miRNAs	Animal models	Cells	Targets/Regulators	Signaling pathways	Function	References
Meg3	268 adult male Sprague–Dawley rats	HMEC-1	NICD, Hes-1, and Hey-1	Notch Pathway	Meg3 inhibits brain lesions, promotes neurological outcomes and induces angiogenesis after IS	Liu et al., 2017
LncRNA-1810034E14Rik	C57BL/6 mice	primary microglial cells	−	NF-κB pathway	1810034E14Rik upregulation decreased the expression of inflammatory cytokines in IS animal and inhibited the microglial cells	Qu et al., 2019
HOTTIP	C57BL/6 mice	Primary cortical neurons	miR-143	miR-143/hexokinase 2 pathway	HOTTIP expression reduced ischemic injury and attenuated glycolytic metabolism in neurons	Liang et al., 2018a
Lnc-M64384, Lnc-MRAK013682, Lnc-MRAK051099	Wistar rats	Neural progenitor cells (NPCs)	−	−	Theses lncRNA may use as an therapy for amelioration of neurological functions.	Liu et al., 2019
lncRNA Rian	C57BL/6 mice	N2a cell line (mouse)	miR-144-3p	Rian/miR-144-3p/GATA3 signaling	Rian inhibits cell apoptosis from cerebral ischemia-reperfusion injury by Rian/miR-144-3p/GATA3 signaling	Yao et al., 2020
miR-10b-3p and miR-217-5p	Brain of 10 Rat MCAO model and 10 controls	−	PPFIA1, SLC7A1, NTRK2, KDM48	Ras signaling pathway, cGMP-PKG signaling pathway, phospholipase D signaling pathway	These miRNAs may control cell proliferation and apoptosis via the cGMP-PKG signaling pathway	Duan et al., 2019
miR-424	plasma and ipsilateral brain tissue from C57/BL6 mice	BV2 microglial cell	CDC25A, cyclin D1, and CDK6	−	miR-424 suppresses neuronal apoptosis and microglia activation	Zhao et al., 2013
miR-126-3p and miR-126-5p	ICR mice	−	SPRED1, VEGFA, and p-Raf-1	MAP kinase pathway, VEGFA/SPRED1/raf-1 signaling pathway	miR-126-3p reduces the OGD/R-induced apoptosis and enhances cell survival.	Xiao et al., 2020
miRNA-126	60 ICR mice	HUVECs	PTPN9	AKT and ERK signaling pathways	miRNA-126 reduces brain atrophy size and enhances neurobehavioral function.	Qu et al., 2019
miR-22	16 SD rats	rat pheochromo- cytoma cell line	TNF-α, IL-1β, IL-6, IL-18, MIP-2 and PGE2	p38 MAPK pathway	miRNA-22 inhibits the inflammatory factors *in vitro*.	Dong et al., 2019
miR-652	SD rats	SH-SY5Y cell lin	NOX2	ROS pathway	miR-652 inhibited NOX2 expression, reduced NOX activity and ROS level and enhanced apoptosis	Zuo et al., 2020
miR-3552	blood and brain specimens from 7 brain specimens from MCAO rats and 5 brain specimens from sham-operated rats	−	CASP3	apoptosis pathway	miR-3552 might regulate apoptosis by CASP3	Li et al., 2020
miR-103	SD rats	HUVECs	VEGF	−	miR-103 inhibits the increase of tube length and the migration of cells and ischemic stroke angiogenesis, and enhances infarction volume	Shi et al., 2018
miR-195	SD rats	cerebral cortex cells RCCNC	KLF5	JNK signaling pathway	miR-195 upregulation suppresses cerebral infarction, loss of neuronal cells, and induces synaptic plasticity	Chang et al., 2020
miR-122	Blood specimens from SD rats	−	Vcam1, Nos2, Pla2g2a	granulocyte/agranulocyte adhesion and diapedesis, leukocyte extravasation, eicosanoid signaling and atherosclerosis signaling	miR-122 upregulation enhances stroke outcomes.	Liu da et al., 2016
miR-579-3p	brain tissue from SD rats	neurons	NRIP1	NF-êB pathway	miR-579-3p has neuroprotective effect and reduces inflammation and apoptosis.	Jia et al., 2020
miR-7a-5p	spontaneously hypertensive rats, C57BL/6 mice	PC12 cells	α-Syn	−	miR-7a-5p improved ischemic brain damage.	Kim et al., 2018
miR-219	Serum and brain tissue from Wistar rats	−	NMDA	−	miR-219 modulated ischemia by NMDA.	Silva et al., 2017
miR-99a	C57BL/6 mice	neuro-2a cells	cyclin D1 and CDK6	−	miR-99a decreased neuronal injury after cerebral I/R.	Tao et al., 2015
miR-126	SD rats	adipose derived stem cells (ADSCs)	−	−	miR-126 induced neurogenesis and vasculogenesis, and suppresses microglial activation and inflammatory response after ischemic stroke.	Geng et al., 2019
miR-130a-3p	MCAO/R mice	SH-SY5Y and N2a cells	DAPK1	H19/miR-130a-3p/DAPK1 axis	miR-130a-3p controls apoptosis in SH-SY5Y and N2a cells as well as on cerebral damage by I/R.	Feng et al., 2021

## Discussion

A wealth of information about the role of non-coding RNAs in the development of IS has been obtained from combination of RNA-sequencing assays and bioinformatics assays such as GO, KEGG pathway enrichment assays and network analyses. These kinds of studies not only exhibited dysregulation of these transcripts, but also provided mechanistical insights about their route of actions. Generally, non-coding RNAs might participate in the pathophysiology of IS through different routes. As a number of differentially expressed lncRNAs between IS patients and healthy controls map to genomic loci near IS-associated genes, regulation of gene expression through *cis*-acting modes is a possible route. Another possible mechanism of contribution of lncRNAs in the pathology of IS is their ceRNA role. MEG3/miR-424-5p, KCNQ1OT1/miR-200a and MALAT1/miR-205-5p are few examples of interplay between lncRNAs and miRNAs in the context of IS.

Aberrant expression of non-coding RNAs in IS patients might be due to the presence of a number of genomic variants within the coding genes as demonstrated for ANRIL lncRNA. This lncRNA is among the mostly assessed lncRNAs in IS. However, the results of all studies are not consistent in this regard. Such inconsistency might be due to phase of sampling during the course of IS or the presence of other confounding parameters. The presence of lncRNAs in the serum specimens and exosomes extracted from these specimens facilitates diagnosis of IS and its clinical variants using this noninvasive route of sampling.

MicroRNAs contribute in the pathogenesis of IS through modulation of genes implicated in the atherosclerosis or inflammatory responses. Exosomal miRNAs might affect communication between several types of cells including endothelial and smooth muscle cells. IS-related circulating miRNAs might hypothetically exert similar functions. Yet, this hypothesis should be judged in upcoming studies. Peripheral expression of miRNAs can be used to differentiate IS patients from healthy subjects or IS patients from other related conditions such as HS. Moreover, their signature might predict recovery from IS-related clinical signs.

The observed sex-biased pattern of differentially expression of lncRNAs (Dykstra-Aiello et al., 2016) might determine different pathogenic processes for the evolution of IS among men and women which should be further examined. Moreover, a number of investigations have displayed specific lncRNA signatures at certain time points following IS, demonstrating the specific roles of lncRNAs in each step of pathogenic processes following IS.

In spite of conduction of various functional studies to unravel the role of non-coding RNAs in IS, therapeutic application of these transcripts have not been clarified. Therefore, future investigation should appraise the possibility of using these transcripts as therapeutic targets in IS. Another limitation of most of mentioned studies is their relatively small sample sizes and lack of simultaneous appraisal of exposures and outcomes in cross-sectional studies. Application of non-coding RNAs as therapeutic targets for IS has faced some challenges in terms of safe delivery of the drug to specific targets, avoidance of off-target effects and determination of best time for intervention. This filed is still in its infancy.

## Author Contributions

SG-F wrote the manuscript and revised it. MT designed the study and supervised it. NA, ZS-F, and BH collected the data and designed the figures and the tables. All authors approved the manuscript.

## Conflict of Interest

The authors declare that the research was conducted in the absence of any commercial or financial relationships that could be construed as a potential conflict of interest. The reviewer RE declared a shared affiliation with several of the authors, SG-F, ZS-F, and NA, to the handling editor at the time of the review.

## Publisher’s Note

All claims expressed in this article are solely those of the authors and do not necessarily represent those of their affiliated organizations, or those of the publisher, the editors and the reviewers. Any product that may be evaluated in this article, or claim that may be made by its manufacturer, is not guaranteed or endorsed by the publisher.

## Abbreviations

AF: atrial fibrillation
AUC: area under curve
ceRNAs: competing endogenous RNAs
GO: gene ontology
HS: hemorrhagic stroke
hs-CRP: high-sensitivity C-reactive protein
IS: Ischemic stroke
LAA: large-artery atherosclerosis
lncRNAs: long non-coding RNAs
MCAO: middle cerebral artery occlusion
miRNAs: microRNAs
NIHSS: NIH Stroke Scale
PBMCs: peripheral blood mononuclear cells
ROC: receiver operating characteristic
TIA: transient ischemic attack.
